# 7-Chloroquinolin-4-yl Arylhydrazone Derivatives: Synthesis and Antifungal Activity

**DOI:** 10.1100/tsw.2011.141

**Published:** 2011-07-28

**Authors:** Auri R. Duval, Pedro H. Carvalho, Maieli C. Soares, Daniela P. Gouvêa, Geonir M. Siqueira, Rafael G. Lund, Wilson Cunico

**Affiliations:** ^1^ NuQuiA—Núcleo de Química Aplicada, Centro de Ciências Químicas, Farmacêuticas e de Alimentos (CCQFA), Universidade Federal de Pelotas, Campus Universitário, Pelotas, RS, Brazil; ^2^ Laboratório de Microbiologia Oral, Faculdade de Odontologia, Universidade Federal de Pelotas, Pelotas, RS, Brazil

**Keywords:** antifungal drugs, hydrazones, thiazolidinones, microbial sensitivity tests

## Abstract

Fifteen 7-chloro-4-arylhydrazonequinolines have been evaluated for their in vitro antifungal activity against eight oral fungi: *Candida albicans, C. parapsilosis, C. lipolytica, C. tropicalis, C. famata, C. glabrata, Rhodutorula mucilaginosa*, and *R. glutinis*. Several compounds exhibited minimum inhibitory concentration (MIC) and minimum fungicidal concentration (MFC) activities comparable with the first-line drug fluconazole. These results could be considered as an important starting point for the rational design of new antifungal agents.

## INTRODUCTION

*Candida albicans* is a yeast-like dimorphic fungus that can be found as part of the normal flora in the mouth, feces, and vaginal secretions of adults[1]. The progression of the infection in mucous membranes was referred as a process that depends on the host defense and on the ability of *Candida* spp. to overcome this defense mechanism[2]. Chronic atrophic (erythematous) candidiasis, also known as “denture stomatitis”, is a clinical presentation of oral candidiasis, especially in the elderly and particularly in those who wear dentures and do not present good mouth care regimen[3]. *Rhodotorula* spp. of the Criptococcaceae family are common airborne organisms that may be present on the skin and in sputum, urine, and feces. Furthermore, they are commonly isolated from patients with denture stomatitis and have been implicated as an infrequent cause of infections, such as septicemia, endocarditis, meningitis, and peritonitis. To date, most infections caused by *Rhodotorula* spp. have been associated with intravenous catheters and with patients who have solid tumors, lymphoproliferative diseases, chronic renal failure, diabetes, endocarditis, pulmonary diseases, and AIDS. Because of the intrinsic resistance of *Rhodotorula* spp. to the triazoles and echinocandins, patients that receive fluconazole and caspofungin are susceptible to develop breakthrough *Rhodotorula* fungemia[4].

Unfortunately, the use of the limited numbers of antifungal agents to treat fungal infections has led to the development of drug resistance. Hence, the development of new antifungal drugs should be encouraged.

Hydrazones are an important intermediate group for the synthesis of a large number of compounds, especially heterocyclic thiazolidinones. Thiazolidinones are five-membered heterocycles with one nitrogen atom, one sulfur atom, and a carbonyl group, which have valuable biological activities[5]. The literature shows the potential antimicrobial activity of hydrazones[6,7,8,9], and due to synthetic and biological versatility of such compounds, they are attractive target compounds for new drug development[10].

The 7-chloroquinoline moiety was extensively studied, mainly for its antimalarial properties[11,12]. In this context, we studied the application of 7-chloro-4-hydrazinoquinoline as a precursor to the synthesis of (trifluoromethyl-1H-pyrazol-1-yl)chloroquine analogues and the biological study of such heterocycles against *Plasmodium falciparum*[13]. Recently, some 7-chloro-4-hydrazonequinoline derivatives have also been investigated as antitubercular agents[14,15] and in the course of our investigations on the chemistry of five-membered heterocyclic thiazolidinones[16,17,18], the aims of this work were the synthesis and antifungal activities of 7-chloro-4-arylhydrazonequinolines **4a–o** and their application as precursors to the synthesis of thiazolidinones.

## RESULTS AND DISCUSSION

The synthetic route for the preparation of 7-chloro-4-arylhydrazonequinoline derivatives is summarized in Scheme 1. The reaction of 4,7-dichloroquinoline with hydrazine hydrate (80%) in reflux of absolute ethanol for 4 h gave 7-chloro-4-hydrazinoquinoline **2** in good yields[19]. In our study, we initially isolated the hydrazone intermediates **4a–o**, refluxing the 7-chloro-4-hydrazinoquinoline **2** with arenealdehydes **3a–o** in toluene with water removal using a Dean-Stark apparatus (Scheme 1). After the reaction time, the solvent was removed and the products were obtained in near quantitative yields (92–98%). Hydrazones **4a–o** were identified by $^1$H NMR and by melting points, and are in agreement with the literature data[14,20].

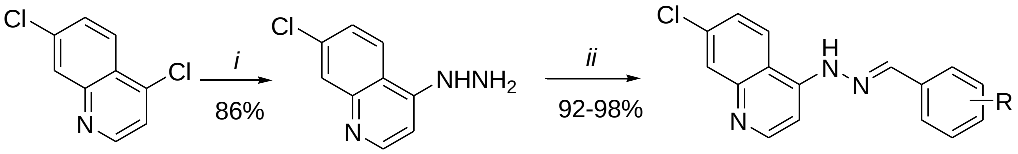

**1** **2** **4a-o**

*i*: $NH_2NH_2.H_2O$ (80%), MeOH, reflux, 4h

*ii*: arenealdehyde **3a-o**, toluene, reflux, 3h

**SCHEME 1.**

Subsequently, the reaction of hydrazones **4a–o** with an excess of mercaptoacetic acid was carried out in reflux of toluene for long reaction times (24–72 h). To our surprise, the expected heterocyclic thiazolidinones were not obtained and the precursors were fully recovered. A possible explanation is the low solubility of hydrazones in toluene; however, even when we carried out the reaction in dimethylsulfoxide (DMSO) at 120°C for long time periods, no thiazolidinones were formed. Recently, we published an improved methodology to synthesize thiazolidinones from hydrazones using only mercaptoacetic acid as solvent[21]. However, in this condition and even using ultrasound irradiation[22], the desired heterocycle was not observed by TLC or GC.

Table 1 shows the antifungal activities of compounds **4a–o** expressed as minimum inhibitory concentration (MIC) and minimum fungicidal concentration (MFC) against eight *C. albicans* strains. Two compounds (**4a** and **4o**) exhibited antifungal activity in comparison with the standard drug fluconazole and the hydrazone **4a** (R = 2-F) showed the highest activities (MIC = 25 μg/mL and MFC = 50 μg/mL). For hydrazones **4b–n** the MIC and MFC values were higher than 200 μg/mL, thus considered inactive. These results might indicate that the presence of a small fluorine atom on the 2-position of the benzene ring seems to be important for the antifungal activity since the chlorine atom (**4d**) did not show antifungal activity.

**TABLE 1**
**Antifungal Activity of Hydrazones 4a–o against Eight *C. albicans* Strains (μg/mL)**

| | R | MW | *C. albicans* | | | R | MW | *C. albicans* | |
|---|---|---|---|---|---|---|---|---|---|
| | | | MIC | MFC | | | | MIC | MFC |
| **4a** | 2-F | 299 | 25 | 50 | **4i** | 3-$OCH_3$ | 311 | >200 | >200 |
| **4b** | 3-F | 299 | >200 | >200 | **4j** | 4-$OCH_3$ | 311 | >200 | >200 |
| **4c** | 4-F | 299 | >200 | >200 | **4k** | 2-CN | 306 | 100 | >200 |
| **4d** | 2-Cl | 315 | >200 | >200 | **4l** | 3-CN | 306 | 84 | 100 |
| **4e** | 2-OH | 297 | >200 | >200 | **4m** | 4-CN | 306 | >200 | >200 |
| **4f** | 3-OH | 297 | >200 | >200 | **4n** | 3-$NO_2$ | 326 | >200 | >200 |
| **4g** | 4-OH | 297 | 100 | 100 | **4o** | 4-$CH_3$ | 295 | 100 | 60 |
| **4h** | 2-$OCH_3$ | 311 | >200 | >200 | Fluconazole | | 306 | 1–16 | 1–16 |

The values are the mean of at least three determinations.

We explored in more detail the antifungal potential of the hydrazones **4a, 4g, 4l,** and **4o**. These four compounds were also tested against the following fungi: *C. parapsilosis* (three strains); *C. lipolytica* (two strains); and *C. tropicalis, C. famata, C. glabrata, Rhodutorula mucilaginosa*, and *R. glutinis* (one strain). Compound **4a** showed good activity (MIC = 50 μg/mL and MFC = 50 μg/mL) against four fungi (Table 2); however, this compound was cytotoxic on mouse fibroblast cells. At a concentration of 50 μg/mL, cell viability was reduced by 92% after 24 h of incubation (Table 3). Hydrazones **4g** and **4l** did not show good antifungal activity (Table 2) and they were also cytotoxic (Table 3). Hydrazone **4o** (R = $CH_3$) showed the best MIC and MFC values (40 μg/mL) for all fungi, with the exception of *C. tropicalis* (Table 2), and also showed low cytotoxicity (82% of cell viability at 50 μg/mL after 24 h, Table 3). The MIC value against *R. glutinis* of the most active compound **4o** was comparable to that of fluconazole (MIC = 32 μg/mL).

## CONCLUSION

The intermediate hydrazones **4a–o** were easily synthesized in excellent yields. The corresponding thiazolidinones were not obtained from hydrazones; however, hydrazones **4a–o** possess potential biological applications. In this work, we explored the antifungal activity of all hydrazones against *C. albicans* and one of them, **4a**, exhibited a MIC value of 25 μg/mL and a MFC value of 50 μg/mL. The compounds **4a, 4g, 4l,** and **4o** were also tested against five other yeasts species of *Candida* and two yeasts species of *Rhodutorula.* The hydrazone **4o** showed the lowest MIC and MFC values (40 μg/mL). The most active compound, **4o**, showed similar antifungal activity against *R. glutinis* when compared to fluconazole. These results could be considered an important starting point to develop new antifungal agents.

**TABLE 2**
**Antifungal Activity of Hydrazones 4a, 4g, 4l, and 4o Expressed as MIC and MFC in µg/mL**

| | R | *R. glutinis* | | *R. mucillaginosa* | | *C. glabrata* | | *C. parapsilosis* | | *C. famata* | | *C. lipolytica* | | *C. tropicalis* | |
|---|---|---|---|---|---|---|---|---|---|---|---|---|---|---|---|
| | | MIC | MFC | MIC | MFC | MIC | MFC | MIC | MFC | MIC | MFC | MIC | MFC | MIC | MFC |
| **4a** | 2-F | 50 | 50 | 50 | 50 | >200 | >200 | 50 | 50 | >200 | >200 | 50 | 50 | 100 | 100 |
| **4g** | 4-OH | >200 | >200 | 87 | 100 | 87 | 100 | 87 | 100 | >200 | >200 | 87 | 100 | >200 | >200 |
| **4l** | 3-CN | >200 | >200 | 84 | 100 | >200 | >200 | 84 | 100 | >200 | >200 | 84 | 100 | 84 | 100 |
| **4o** | 4-$CH_3$ | 40 | 40 | 40 | 40 | 40 | 40 | 40 | 40 | 40 | 40 | 40 | 40 | 80 | 80 |
| Fluconazole | | 32 | 32 | 8 | 8 | 2 | 2 | 2 | 2 | 4 | 4 | 8 | 8 | 2 | 2 |

The values are the mean of at least three determinations.

**TABLE 3**
**Cytotoxicity of Compounds 4a, 4g, 4l, and 4o against Mouse Fibroblasts 3T3/NIH Cell after 24 and 48 h (µg/mL)**

| | 24 h (% of Cell Viability) | | | | 48 h (% of Cell Viability) | | | |
|---|---|---|---|---|---|---|---|---|
| | 100 | 50 | 25 | 12.75 | 100 | 50 | 25 | 12.75 |
| **4a** | 9 | 8 | 10 | 65 | 7 | 7 | 7 | 19 |
| **4g** | 7 | 33 | 20 | 38 | 4 | 22 | 19 | 28 |
| **4l** | 9 | 8 | 14 | 18 | 2 | 2 | 2 | 4 |
| **4o** | 51 | 82 | 85 | 95 | 45 | 79 | 75 | 90 |

The values are the mean of at least three determinations.

## Experimental

Unless otherwise indicated, all reagents and solvents were used as obtained from commercial suppliers without further purification. $^1$H spectra were recorded on a Bruker DRX 400 spectrometer ($^1$H at 400.14 MHz) in DMSO containing tetramethylsilane as an internal standard. Melting points were determined using open capillaries on a Fisatom model 430 apparatus and are uncorrected. The purity of compounds were confirmed by GC analyses in a Shimadzu Gas Chromatograph GC-2010 apparatus column HP-1 (column I.D., 0.25 mm; column length, 30 m; column head pressure, 14 psi; program: $T_0$ = 50°C; $t_0$ = 2.0 min; rate 16.0°C/min; $T_f$ = 250°C; $t_f$ = 10.0 min; Inj. = 250°C; Det. = 270°C).

## General Procedure for the Synthesis of 7-Chloro-4-Arylhydrazonequinolines 4a-o

A mixture of 7-chloro-4-hydrazinoquinoline **2** (2 mmol) and the appropriate arenealdehydes **3a–o** in toluene was heated at 110°C with a Dean-stark trap until reaction was complete, as shown by TLC (about 3 h). After reaction time, the solvent was removed and the solid was washed with cold ethyl ether, leading to the hydrazones **4a–o** without any further purification.

## Antifungal Activity

The antifungal assay was carried out following the CLSI M27-A3 (2008) method[23]. Hydrazones **4a–o** were tested against 18 strains of eight species of oral yeasts: *Candida albicans* (8), *C. parapsolisis* (3), *C.*

*lipolytica* (2), *C. tropicalis* (1), *C. famata* (1), *C. glabrata* (1), *Rhodutorula mucilaginosa* (1), and *R. glutinis* (1). The strains were obtained from the Laboratory of Oral Microbiology, Pelotas Dental School, at Federal University of Pelotas, Brazil. This study was approved by the Ethics Committee from Pelotas Dental School (Document nº. 036/2006).

### *Sample Preparation*

The fungal suspensions were prepared by including a 24-h, previously activated strain in a test tube with 5 mL of sterile normal saline, adjusted to 0.5 McFarland scale ($1.5 \times 10^8$ microorganisms/mL). The final inoculum contained $3.75 \times 10^4$ yeast cells/mL.

### *The Test*

Ten dilutions of compounds **4a–o** were prepared with concentrations ranging from 0.2 to 104 µg/mL in DMSO. The fungal suspension was transferred in aliquots of 100 µL into each well of the sterile plates containing 100 µL of the compound dilution. Wells 11 and 12 were, respectively, the negative control (200 µL of RPMI culture medium) and the positive control (100 µL of RPMI culture medium + 100 µL of the fungal suspension). The plates were incubated at 37ºC for 96 h. The lowest concentration of the drug that produced the inhibition of the growth of the yeast was identified as the MIC value of the drug. To evaluate the MFC, 20 µL of each solution that was considered a MIC concentration were plated onto a 9-mm plate containing Sauboraud Dextrose Agar[24]. The MFCs were defined as the lowest drug dilutions that did not yield growth of yeast colonies (99.88% killing activity) after 24 h of aerobic incubation at 37ºC. Fluconazole was used as reference compound[25,26].

## Cytotoxicity Assay

The culture medium was obtained from GIBCO (Grand Island, NY, USA). All others reagents were from Sigma (Sigma Chemical Company, St. Louis, MO, USA). Compounds **4a**, **4g**, **4l,** and **4o** were diluted in Dulbecco’s modified Eagle’s medium (DMEM). Series of dilutions were prepared, ranging between 12.75 and 100 µg/mL of a previous solution in DMSO. The cell culture medium was DMEM supplemented with 10% fetal bovine serum, 2% l-glutamine, penicillin (100 U/mL), and streptomycin (100 mg/mL). Mouse fibroblasts of the 3T3/NIH immortalized cell line were maintained as a stock culture in DMEM and incubated at 37°C in a humidified atmosphere of 5% $CO_2$ in air until subconfluency.

### *Cytotoxicity Assay (MTT Assay)*

The 3-(4,5-dimethylthiazol-2-yl)-2,5-diphenyltetrazolium bromide (MTT) assay was used to assess cell metabolic function by mitochondrial dehydrogenase activity. Mouse fibroblasts 3T3/NIH ($2 \times 10^4$/well) were maintained in DMEM in 96-well plates for 24 h. Cytotoxicity produced by different dilutions of hydrazone derivatives was assessed in a 24- and 48-h cell-exposure time. After removing the test product, cells were washed with phosphate-buffered saline (PBS). Then 200 µL of medium with 20 µL of MTT solution (5 mg/mL MTT in PBS) was added to each well. After 5 h of incubation at 37°C in darkness, the blue formazan precipitation was extracted from the mitochondria using 200 µL/well DMSO on a shaker for 5 min at 180 *g*. The optical density at 540 nm was determined spectrophotometrically. All experiments were performed three times.

## ACKNOWLEDGMENTS

The authors thank Universidade Federal de Pelotas (UFPel) and Conselho Nacional de Desenvolvimento Científico e Tecnológico (CNPq) (proc. 305314/2009-2) for the financial support of the research. Coordenação de Aperfeiçoamento de Pessoal de Nível Superior (CAPES) and Fundação de Amparo à Pesquisa do Estado do Rio Grande do Sul (FAPERGS) are also acknowledged.
